# Incidence of parvovirus B19 among Hungarian blood donor population during COVID-19 restrictions and the subsequent B19 epidemic of 2024

**DOI:** 10.1017/S0950268825100861

**Published:** 2026-01-02

**Authors:** László Kokavecz, Zita Sohajda, Péter Dávid, Anikó Stágel, Klára Baróti-Tóth, Sándor Nagy, Melinda Paholcsek, Réka Sohajda

**Affiliations:** 1 Nucleic Acid Testing Laboratory, Hungarian National Blood Transfusion Service, Hungary; 2 Department of Otorhinolaryngology, Hetényi Géza Clinic, Szolnok, Hungary; 3 Complex Systems and Microbiome-innovations Centre, University of Debrecen, Hungary; 4 Hungarian National Blood Transfusion Service, Hungary

**Keywords:** blood transfusion-associated infections, epidemiology, parvovirus, blood donor screening, PCR detection, COVID-19 restrictions, viral transmission

## Abstract

Nationwide screening for parvovirus B19 among blood donors in Hungary has been conducted since 2019. Although B19 is primarily transmitted via the respiratory route, transfusion-related transmission also occurs. This study investigated the impact of COVID-19–related restrictions on B19 incidence. Between January 1 2019 and December 31 2024, a total of 2,043,119 blood donations were screened for B19 DNA using PCR, and the study period was divided into six epidemiological phases.

During the pre-restriction period (Phase I), B19 incidence was relatively low (0.87/10,000 donations). Following the introduction of COVID-19 restrictions (Phase II), highly viremic donations were not detected. Incidence gradually returned in Phase III (0.22/10000) and increased in Phase IV (1.96/10000), suggesting a minor outbreak. A marked surge in December 2023 (23.03/10000) initiated a nationwide epidemic, peaking in March–April 2024 (46.01/10000), before declining by August (Phase VI; 0.54/10000).

COVID-19 restrictions substantially reduced B19 transmission and may have led to increased population susceptibility. This likely contributed to the unusually intense B19 epidemic observed in 2024, which was considerably more severe than contemporaneous outbreaks reported in other countries.

## Introduction

### Parvovirus B19

Parvovirus B19 (B19) is a small, non-enveloped DNA virus discovered in a healthy donor by Yvonne Cossart in 1975 [1]. B19 can cause a variety of diseases, such as erythema infectiosum (fifth disease), aplastic crisis, chronic pure red blood cell aplasia, foetal hydrops, foetal death, and so on [2]. B19 infection is most dangerous in patients with altered rates of red blood cell substitution, such as those with sickle cell disease or beta-thalassemia, where the infection can progress to extensive bone marrow necrosis, which may be fatal [3, 4]. Most infections occur in childhood and cause fifth disease, which results in a mild rash and the formation of protective antibodies [5]. In the adult population, most infections are asymptomatic or mild, depending on the immune status of the host [6].

### Transmission of parvovirus B19

The main transmission route of B19 is presumably the respiratory tract [7]. In temperate climates, infections are more common during the late winter, spring, and summer months [8], and Kooistra et al. observed a 3–4-year epidemic cycle in their study [9]. Preventing community acquisition of B19 infection is not practical, as transmission primarily occurs via respiratory droplets, usually from person to person. In most cases, approximately 1 week after inhaling infected droplets, viremia is observed, associated with nonspecific symptoms such as fever, malaise, and myalgia. Additionally, B19 can be transmitted in plasma, erythrocyte and platelet concentrates, bone marrow, stem cells, serum albumin, and other blood products. The incidence of positive blood products is highest during peak community transmission of B19 [9]. Since no vaccine or antiviral treatment is available, the most effective way to prevent transfusion-transmitted infections is laboratory screening of blood products [10].

### Detection of parvovirus B19

Since the symptoms of B19 infection are often immune-mediated, detection of IgM and IgG antibodies is the primary method for diagnosing B19 in the normal host [11, 12]. In recent years, several polymerase chain reaction (PCR)-based assays have been developed, making the detection of B19 infection more precise and commercially viable [12]. The International World Health Organization (WHO) standard for B19 nucleic acid amplification techniques has been established, which is especially important for screening blood products, making it the most widely used method of detection worldwide [13].

To reduce the risk of transfusion-transmitted infections, the Hungarian National Blood Transfusion Service introduced B19 screening for whole-blood donations in 2019.

### SARS-CoV-2

The coronavirus disease 2019 (COVID-19), caused by the novel coronavirus known as SARS-CoV-2, was first reported in China in late December 2019 and quickly spread worldwide [14]. SARS-CoV-2 is an enveloped, positive-sense, single-stranded RNA beta-coronavirus [15]. As of 2 January 2025, there were 777,185,539 confirmed cases of COVID-19, and the total number of deaths due to COVID-19 worldwide was 7,081,008 [16].

### Transmission of SARS-CoV-2

SARS-CoV-2 can spread through both direct means (droplet and human-to-human transmission) and indirect contact (contaminated surfaces and aerosol contact). Human-to-human transmission of the virus primarily occurs through coughing, sneezing, or even talking and singing, via the spread of respiratory droplets or aerosols [17].

### Introduction of COVID-19 restrictions in Hungary

The first confirmed case of SARS-CoV-2 infection in Hungary was reported on 4 March 2020. From then on, rules such as mask-wearing were gradually tightened, and a number of restrictions were implemented, including limiting opening hours, travel, public gatherings, and visitations to public places and healthcare facilities [18, 19]. From June to October, certain restrictions were eased, but in November, the government reimplemented and tightened most of them, even introducing a mandatory curfew [20, 21]. On 26 December 2020, health workers began receiving the vaccine.

Starting in April 2021, as the number of vaccinated people reached certain thresholds, restrictions began to ease in increments [22]. At the end of May, the curfew was lifted, wearing masks outdoors was no longer mandatory, and restrictions on opening hours were abolished [23]. By the beginning of July 2021, most of the restrictions had been lifted [24].

The aim of this study is to analyse the effects of COVID-19 restrictions on the incidence of B19 infections among Hungarian blood donor population and to examine whether these restrictions had any impact on the subsequent B19 epidemic of 2024.

## Materials and methods

### Blood collection

Blood donations were collected at blood centres and mobile units across Hungary. Donors who did not meet predefined criteria were rejected from donation. The enrolment criteria included the following parameters: (I) donor age must be at least 18, but not exceeding 65 (60 for first-time donors); (II) the donor’s body weight must exceed 50 kg; (III) the donor’s haemoglobin level must be between 125 g/l and 165 g/l for female donors, and 135 g/l and 180 g/l for male donors; (IV) the donor’s systolic blood pressure must be between 101 and 180 mmHg; (V) the donor’s diastolic blood pressure must not exceed 100 mmHg; (VI) the donor’s pulse must be between 50 and 100 beats per minute. Donors were also interviewed about their medical history and potential high-risk behaviour. Based on this information, individuals with recent tattoos, surgeries, transfusions, etc., were also rejected from donation.

Mandatory nucleic acid testing (NAT) for B19 in Hungary began in January 2019 at the NAT Testing Laboratory of the Hungarian National Blood Service in Budapest. Since then, until January 2025, every whole-blood donation has been screened for B19 infection.

### Laboratory equipment

Pooling of the sample tubes was performed using the Hamilton MICROLAB STAR IVD pipettor (Hamilton Company, USA) by pipetting the supernatant plasma. Extraction, purification, and amplification of the nucleic acids were all performed using the Cobas 6800 instrument (Roche Molecular Systems, USA) with the DPX testing kit.

### Testing method and evaluation

Testing was conducted in minipools of 96 samples (MP96), where 40 μl of plasma from each of the 96 samples was pipetted into a pool collection tube. In the case of a reactive MP96, the pool of 96 samples was divided into 16 secondary pools of six samples (SP6). A SP6 tube contained 200 μl of plasma from each of the six samples. When a reactive SP6 was identified, the respective samples from the SP6 were individually tested to determine the origin of the reactive donation (IDT). This procedure remained unchanged throughout the study period. PCR cut-off values were set at 400, 6400, and 38,400 IU/ml for MP96, SP6, and IDT, respectively.

Products with a B19 DNA virus load equal to or above the aforementioned cut-off values were discarded. MP96 or SP6 pools with a virus load below the cut-off values were not resolved, and donations included in these pools were designated as non-reactive and released for transfusion. If the reactive pool was not resolved, it was considered a B19-infected sample in this study. However, we acknowledge that an unresolved reactive pool might have included multiple positive samples, particularly during epidemic periods. Pools with titre values lower than 40 IU/ml could not be quantified by the test reagent kit and were not considered reactive in this study.

## Results

The study period, from January 2019 to December 2024, was divided into six phases based on the dates when COVID-19 restrictions were in effect and whether there was any unusual increase in B19 incidence: *Phase I*: The period before the restrictions were implemented; *Phase II*: The period during which the restrictions were in effect; *Phase III*: The period after the restrictions were lifted; *Phase IV:* the period of a minor B19 outbreak in Hungary; *Phase V*: the period of the B19 epidemic in Hungary. *Phase VI*: the period after the number of highly viremic samples decreased to pre-epidemic levels.

Since COVID-19 restrictions were implemented gradually starting in March and April 2020, it is difficult to determine a precise end date for Phase I. However, since mandatory mask-wearing in public places was only introduced at the end of April, we concluded that Phase I ended in April 2020. Similarly, it is difficult to determine the end date of Phase II, as the final easing of restrictions occurred between April and June 2021. However, the largest restrictions were lifted in May, so we concluded that Phase II spanned from May 2020 to May 2021. Therefore, Phase III began in June 2021. At the start of March 2023, a moderate increase in the incidence of B19 was observed, indicating the end of Phase III. The increase observed in March 2023 lasted until November 2023; therefore, we designated this minor outbreak as Phase IV. In December 2023, the number of B19-positive samples started to increase sharply. Since the Hungarian National Public Health Centre does not monitor the spread of B19, there was no official announcement marking the start of the epidemic. Based on the sharp increase in cases, we concluded that Phase V began in December 2023. In August 2024, although the number of all viremic donations remained well above average, the number of highly viremic donations returned to pre-epidemic levels. Therefore, we concluded that Phase VI, the post-epidemic phase, began in August 2024.

### Phase I

**Figure 1.**
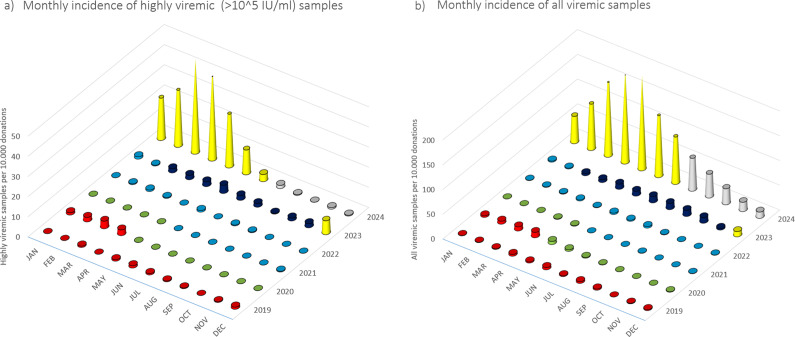
Monthly incidences of highly viremic (>10^5^ IU/ml) (a) and all viremic (b) samples. Bars are colored by phase: red (Phase I), green (Phase II), blue (Phase III), purple (Phase IV), yellow (Phase V), and grey (Phase VI).

**Figure 2.**
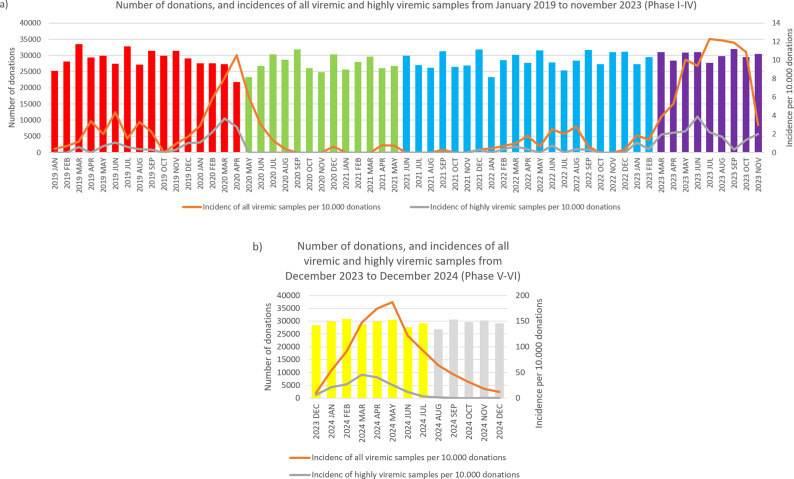
Monthly number of donations and incidence of B19. Bars represent the number of donations each month, color-coded by phase: red (Phase I), green (Phase II), blue (Phase III), purple (Phase IV), yellow (Phase V), and grey (Phase VI). The orange line shows the number of viremic detections per 10 000 donations, while the grey line indicates the incidence of highly viremic samples (>10^5^ IU/ml) per 10 000 donations. Please note that the scaling of the secondary Y-axis differs between panels (a) and (b) due to the large increase in incidence values.

**Figure 3.**
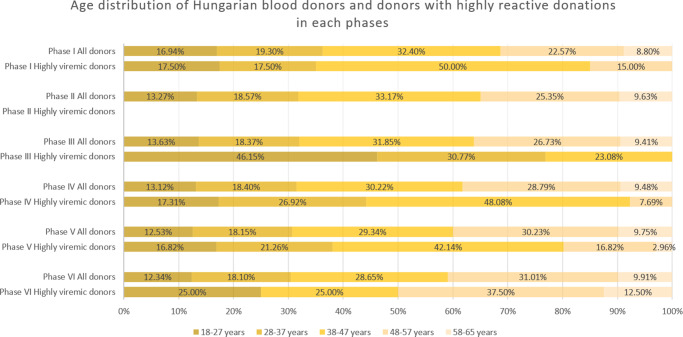
Age distribution of all donations and highly viremic donations (>10^5^ IU/ml) across the study phases. Please note there were no highly viremic donations detected during Phase II.

During January 2019–April 2020, we detected 133 positive cases (Figures 1 and 2a, and Table 1). Of these, 40 samples had a titre value higher than 10^5^ IU/ml and were considered highly viremic (2.89 viremic and 0.87 highly viremic samples per 10,000 donations). Highly viremic cases were concentrated in the 38–47-year age group, while none were detected in donors aged 58–65 years (Figure 3).

**Table 1.** Time periods, total number of donations, number of all and highly viremic detections, and incidence rates of all viremic and highly viremic detections in each phase

Phase	Period	Number of donations	Number of all viremic detections	All viremic detections per 10,000 donations	Number of highly viremic detections	Highly viremic detections per 10,000 donations
Phase I	2019 Jan–2020 Apr	459,619	133	2.89	40	0.87
Phase II	2020 May–2021 May	358,012	33	0.92	0	0.00
Phase III	2021 Jun–2023 Feb	600,266	47	0.78	13	0.22
Phase IV	2023 Mar–2023 Nov	270,572	236	8.72	53	1.96
Phase V	2023 Dec–2024 Jul	234,927	2,592	110.33	541	23.03
Phase VI	2024 Aug–2024 Dec	146,542	327	22.31	8	0.54

### Phase II

Between May 2020 and May 2021, a total of 33 viremic donations were detected, equating to 0.92 viremic samples per 10,000 donations. None of these samples were considered highly viremic (Table 1). Most viremic samples occurred early in this period, followed by an extended phase of minimal activity (Figures 1 and 2a).

### Phase III

In June 2021–February 2023, low-level circulation continued with 47 viremic samples, 13 of which were highly viremic (0.78 viremic and 0.22 highly viremic samples per 10,000 donations; Figures 1 and 2a, and Table 1). Although restrictions were lifted in May 2021, the incidence of B19 infections remained below average throughout the year. Highly viremic donations were mainly from younger donors, whereas none were found among the two oldest age groups (Figure 3).

### Phase IV

**Figure 4.**
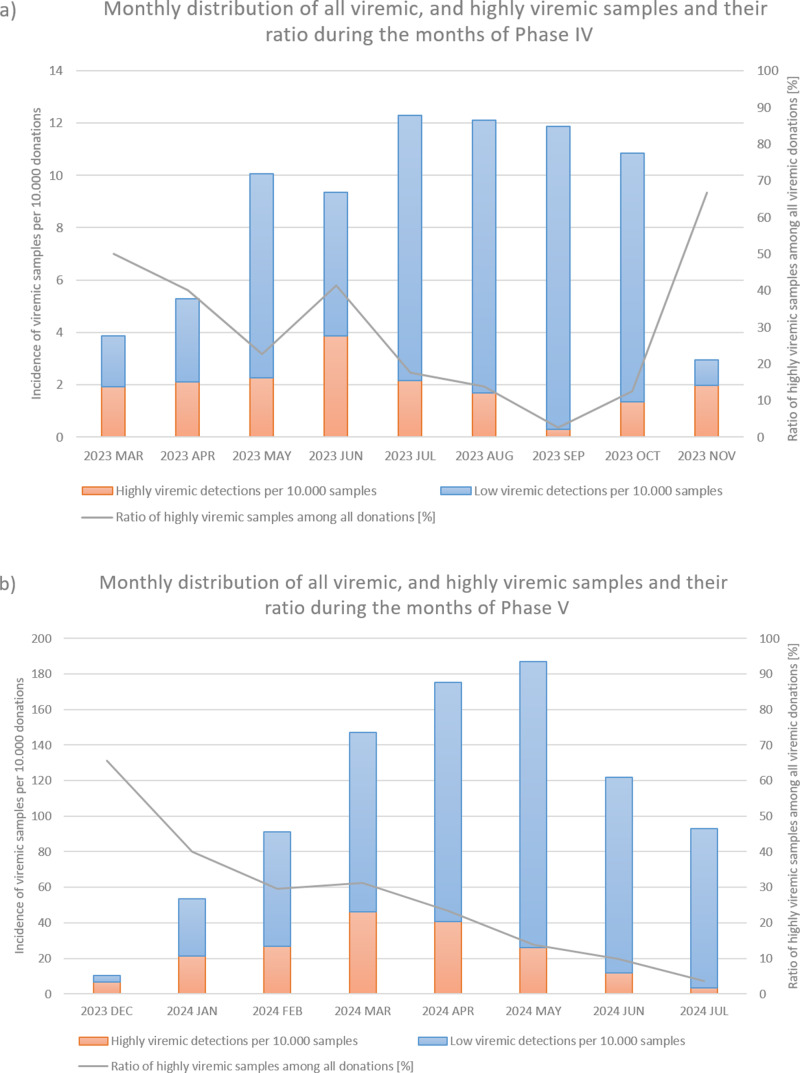
Monthly incidences per 10 000 donations of all viremic samples (red and blue bars combined) and highly viremic samples (>10^5^ IU/ml, represented by the red portion of the bars), along with their respective ratios represented by the grey line during Phase IV (panel a) and Phase V (panel b).

From March to November 2023, incidence increased, with 236 viremic samples, including 53 highly viremic ones (8.72 viremic and 1.96 highly viremic samples per 10,000 donations; Figures 1 and 2a, and Table 1). Since both of these values are significantly higher than in previous periods, this phase was designated as a minor B19 outbreak in Hungary. Donors aged 38–47 years old showed the highest proportion of highly viremic samples, while the two oldest age groups (48–57 and 58–65 years) contributed a substantial proportion of donations, yet only 7.69% of highly viremic cases (Figure 3). The ratio of highly viremic detections to all viremic detections was initially high with 50% and then declined over time, with small peaks in June (outbreak peak) and October–November (possible transition toward Phase V; Figure 4a).

### Phase V

Between December 2023 and July 2024, incidence reached unprecedented levels, with 2,592 viremic cases (110.33 B19-positive samples per 10,000 donations; Figures 1a and 2b, and Table 1). It is worth mentioning that this number might underrepresent the actual incidence, as pool tubes with concentrations under the predetermined thresholds were not resolved and may have contained multiple low-viremic samples. This incidence is 140 times higher than the incidence in Phase III and 12 times higher than the minor outbreak period of Phase IV. There was also a significant increase in highly viremic samples, with 541 detected during this eight-month period (23.03 highly viremic samples per 10,000 donations; Figures 1b and 2b, and Table 1), representing a 106-fold increase compared to Phase III and an almost 12-fold increase compared to the minor outbreak period of Phase IV.

In March, the number of highly viremic samples reached its peak, with 46.01 positives per 10,000 donations, while the number of all viremic detections peaked 2 months later in May with 187.05 detections per 10,000 donations. This lag between the two peaks can most likely be explained by the phenomenon described by Schmidt et al., who reported that previously highly viremic donors still showed signs of detectable B19 viremia months after their initial positive donations [8]. The ratio of highly viremic samples to total viremic samples followed a similar trend to that observed in Phase IV, with a relatively high ratio of 65.52% at the start of the epidemic (Figure 4b), followed by a decreasing trend except for March, when the incidence of highly viremic samples peaked.

Consistent with previous phases, the 38–47-year age group exhibited a disproportionately high rate of highly viremic donations. However, for the first time, highly viremic donations were also detected among the oldest donors (Figure 3).

### Phase VI

From August 2024 to December 2024, viremic incidence decreased, with 327 viremic samples detected (22.31 viremic samples per 10,000 donations). This rate appears lower than during the epidemic period, but higher than in the non-epidemic or the minor outbreak period of Phase IV. Highly viremic samples decreased sharply, with eight detected in this period (0.54 per 10,000 donations), which is comparable to other non-epidemic phases (Figures 1 and 2b, and Table 1). During this period, similar to Phase V, highly viremic donations were also detected among the oldest donors (Figure 3).

## Discussion

Although our data from the pre-pandemic years are limited, the ratio of highly viremic samples observed in Phase I (0.87 per 10,000 donations, January 2019–April 2020) is comparable to ratios reported in other countries for the same period. Plümers et al. reported a ratio of 0.83 per 10,000 donations in Germany between January 2015 and March 2020 [25]. Russcher et al. reported a lower ratio of 0.18–0.6 per 10,000 donations in the Netherlands between 2013 and 2019, although in their study, a sample was considered highly viremic only above a slightly higher threshold of 1.2 × 10^6^ IU/ml [26]. Farcet et al. reported that from June 2018 to February 2020, the average ratio of highly viremic samples in the USA and selected Central European countries (Austria, Czech Republic, and Hungary) was 0.5 and 0.6 per 10,000 donations, respectively – slightly lower than the ratios we observed [27].

Based on our data from Phase II (May 2020–May 2021) and Phase III (June 2021–February 2023), we conclude that the restrictions implemented to reduce the spread of the COVID-19 pandemic had a significant effect on the incidence of parvovirus B19 among Hungarian blood donor population. As soon as stricter restrictions were introduced – such as mandatory mask-wearing in public spaces in May 2020 – the number of viremic detections began to decline. Notably, no highly viremic cases were detected until the restrictions were lifted in May 2021. Even after restrictions were lifted, the incidence remained below average throughout 2021. It took approximately six months after the end of restrictions for incidence levels to return to pre-pandemic values, with an overall rate of 0.22 highly viremic samples per 10,000 donations. Other studies also reported a decrease in B19 incidence during and after the pandemic. Plümers et al. reported an average ratio of 0.06 highly viremic samples per 10,000 donations in Germany from April 2020 to March 2023 [25]. In the Netherlands, Russcher et al. reported ratios between 0 and 0.01 per 10,000 donations between 2020 and 2022 [26]. Farcet et al. examined the period from March 2020 to April 2023 in both Central Europe and the USA, reporting averages of 0.02 and 0.05 highly viremic samples per 10,000 donations, respectively [27].

In Phase IV (March 2023–November 2023), the incidence of both viremic and highly viremic donations was significantly higher than in non-epidemic periods, indicating a minor B19 outbreak in Hungary, with an average of 1.96 highly viremic samples per 10,000 donations. Beginning in December 2023 and lasting until July 2024 (Phase V), an unprecedented increase was observed in the incidence of B19 infections among Hungarian blood donors, with an average of 23.03 highly viremic samples per 10,000 donations and a peak of 46.01 in March 2024. Tóth et al. also reported increased rates of B19 IgM and PCR positivity in the South Transdanubian region of Hungary in 2024 [28]. Most international studies considered these two periods together. Plümers et al. reported that in Germany, between April 2023 and April 2024, the average incidence of highly viremic samples was 5.35 per 10,000 donations, peaking in March 2024 at 21.1 per 10,000 [25]. Farcet et al. examined Central Europe and the USA between May 2023 and May 2024, reporting average incidences of 6 and 1 per 10,000 donations, respectively, with peaks in Central Europe in April 2024 (20 per 10,000 donations) and in the USA in May 2024 at the end of the study period (4 per 10,000 donations) [27]. Compared with these countries, the B19 epidemic in Hungary appeared more intense, with both a higher average incidence and a higher peak. Serological testing of both pregnant women in northern Italy and children under 15 years old in France showed a similar increase in B19-infected cases in early 2024, reporting eightfold and fourfold increases, respectively, compared to previous epidemic periods [29, 30].

Several studies have speculated that COVID-19 restrictions altered immunity patterns and hygiene practices, potentially contributing to this rebound epidemic – similar to the effects observed for other viral infections [31–33]. Based on previously reported data from other European countries and the estimated four-year interepidemic period of B19, Tóth et al. hypothesized that an epidemic wave could be expected around 2020/2021 [28]. Our data from January–April 2020 support this theory, as we observed increased detection rates resembling the start of the 2023 minor outbreak (Figure 2a). However, restrictions and containment measures may have altered B19 transmission dynamics during that period [28].

The age distribution of highly viremic donors varied across study phases. In the first four phases, no cases were detected in the oldest age group (58–65 years), which may reflect prior exposure to B19 and the subsequent development of partial immunity in this cohort. During and after the B19 epidemic (Phases V and VI), however, highly viremic donations were observed in this age group, indicating unusually widespread viral circulation. Notably, following the lifting of restrictions but before the minor outbreak of 2023 (Phase III), the majority of highly viremic donations originated from younger donors. A plausible explanation is that older donors continued to exercise greater caution and maintain preventive practices even after restrictions were lifted, thereby reducing their risk of infection. In contrast, younger donors, who faced lower perceived health risks from COVID-19, may have been less cautious, facilitating B19 transmission within this group. However, the observed increase in the 18–27-year age group was not statistically significant (p = 0.066).

By studying both the minor outbreak of 2023 and the major B19 epidemic of 2024, we observed certain similarities despite their differing intensities. In both cases, the ratio of highly viremic donations to all viremic detections showed a decreasing trend as the epidemic progressed, except during the months when the incidence of highly viremic donations peaked. Since an outbreak of this magnitude caused significant logistical and supply chain challenges, these ratios may serve as useful indicators for predicting the peak and duration of future B19 epidemics. Specifically, monthly ratios above 50% might signal the onset of an epidemic, while smaller increases in the ratio could indicate the peak incidence of highly viremic donations. However, it is important to note that the incidence of all viremic detections may be underestimated in this study, as we did not identify pools with low B19 titres. It is likely that some of these pools contained multiple samples with low B19 concentrations, which could influence the calculated ratios. Moreover, relying solely on the ratio can be misleading in months with very few detections – for example, if only two B19-positive donations are identified and one is highly viremic, the ratio would be relatively high with 50%, even though such a low case number does not reflect epidemic activity. Finally, many other factors influence the dynamics and characteristics of an epidemic (e.g. the COVID-19 restrictions implemented in early 2020, possibly preventing a B19 outbreak, as theorized by Tóth et al. [28]). For this reason, the observed trend should not be regarded as a sole predictor but rather considered alongside other factors, including the current incidence of infections and seasonal context.

## Data Availability

The data used in this study consist of medical records of blood donors and contain sensitive personal health information. Due to ethical considerations and institutional regulations regarding the confidentiality and privacy of medical data, these data cannot be shared publicly.
